# Evaluation of various biomarkers for kidney monitoring during canine leishmaniosis treatment

**DOI:** 10.1186/s12917-017-0956-0

**Published:** 2017-01-23

**Authors:** Luis Pardo-Marín, Silvia Martínez-Subiela, Josep Pastor, Asta Tvarijonaviciute, Juan Diego Garcia-Martinez, Sergi Segarra, José Joaquín Cerón

**Affiliations:** 1 Interdisciplinary Laboratory of Clinical Analysis (Interlab-UMU), Veterinary School, Campus of Excellence Mare Nostrum, University of Murcia, 30100 Espinardo, Murcia Spain; 2 Departament de Medicina i Cirurgia Animals, Universitat Autònoma de Barcelona, 08193 Bellaterra, Barcelona, Spain; 3 R&D Bioiberica SA, Pça. Francesc Macià 7, 08029 Barcelona, Spain

**Keywords:** Urine, Kidney, Immunoglobulin G (IgG), UPC, Creatinine, Symmetric dimethylarginine (SDMA), Gamma glutamyl-transpeptidase (GGT), Retinol-binding protein (RBP)

## Abstract

**Background:**

The objective of this study was to evaluate and compare the evolution of the profile currently recommended by the International Renal Interest Society (IRIS) (sCr, UPC and sSDMA) with a panel of other different kidney biomarkers during treatment for canine leishmaniosis. This panel included three urinary glomerular biomarkers (uIgG, uCRP and uferritin) and three urinary tubular biomarkers (uGGT, uNAG and uRBP).

These biomarkers were measured in two groups of dogs with canine leishmaniosis at IRIS stage I. Group 1: dogs showing proteinuria (UPC > 0.5) before treatment which did not decrease after treatment; Group 2: dogs showing proteinuria before treatment which decreased after treatment.

**Results:**

Group 1 showed no significant changes in any biomarker after treatment. In group 2, among the biomarkers recommended by the IRIS, only UPC showed a significant decrease after treatment. However all biomarkers of glomerular damage showed a significant decrease after treatment, with uIgG/Cr and uCRP/Cr showing the greater decreases. In addition uRBP/Cr and uNAG/Cr showed significant decreases after treatment.

**Conclusions:**

In dogs with leishmaniosis at IRIS stage I that reduced UPC after treatment, there were no significant changes in serum creatinine and sSDMA. However, all the urine biomarkers evaluated with exception of uGGT showed a significant decrease. These decreases were more evident in those markers related with glomerular function, being uIgG/Cr the biomarker more associated with UPC. Further studies involving a larger number of animals and histological analysis of the kidney would be recommended to confirm these findings and evaluate the routine practical use of these urine biomarkers in canine leishmaniosis.

## Background

Canine leishmaniosis is a disease that causes glomerulonephritis and chronic kidney disease (CKD) by immune complex deposition that may lead to end-stage renal failure [1, 2]. Initially, infected dogs can show proteinuria in absence of azotemia. However, in case that glomerular disease progresses, tubulointerstitial lesions, azotemia and end-stage renal failure signs can appear [1]. In the Mediterranean area, canine leishmaniosis is caused by infection with *Leishmania infantum* transmitted by *Phlebotomus* sand fly, and in countries such as Spain the prevalence can reach to 67% in some areas [3].

Serum creatinine (sCr) and urinary protein to creatinine ratio (UPC) are the biomarkers traditionally recommended by the International Renal Interest Society (IRIS) to evaluate and monitor renal damage/dysfunction [4], and are used in the clinical classification of canine leishmaniosis [5]. Recently, the measurement of serum symmetric dimethylarginine (sSDMA) has also been recommended by IRIS. However, there are other biomarkers that can be measured in urine and are useful for kidney evaluation. These biomarkers are very sensitive to evaluate renal damage and additionally have the capacity to quantify and localize the site of renal injury [6, 7]. For example, immunoglobulin G (IgG) and the acute phase proteins C-reactive protein (CRP) and ferritin are high molecular weight proteins to which an intact glomerular barrier is impermeable [8, 9]. Therefore the presence in urine of these proteins would indicate a glomerular damage. In the particular case of IgG it is considered to have a higher specificity than UPC as a marker of the severity of damage to the glomerular capillary wall. This is due to the fact that IgG, contrarily to UPC, is not influenced by tubular lesions [8].

On the other hand, there are proteins in urine that can inform about the existence of tubular dysfunction. For example, retinol-binding protein (RBP) is a low molecular weight protein (21-kDa) synthesized in the liver [10], that can be detected in urine when there is a proximal tubulointerstitial damage impairing reabsorption [8]. In addition, N-aceytyl-β-D-glucosaminidase (NAG) is described as a lysosomal enzyme in the renal tubular epithelial cells that is released when renal damage occurs and gamma glutamyl-transpeptidase (GGT) is related with tubular damage and can identify dogs with tubular proteinuria [11].

To the authors’ knowledge, there are no studies in which the dynamics of the biomarkers recommended by the IRIS during canine leishmaniosis treatment are analysed and compared with other different glomerular and tubular biomarkers.

Therefore, the objective of this study was to evaluate and compare the evolution of the profile currently recommended by the IRIS (sCr, UPC and sSDMA) with a panel of other different kidney biomarkers during treatment for canine leishmaniosis. This panel included three urinary glomerular biomarkers (uIgG, uCRP and uferritin) and three urinary tubular biomarkers (uGGT, uNAG and uRBP).

To the authors’ knowledge this is the first time in which a comprehensive profile of renal biomarkers is evaluated in urine in the treatment of canine leishmaniosis. In addition uRBG and sSDMA will be studied for the first time in dogs with this disease.

The evaluation of these biomarkers could provide new insights about how biomarkers representing different parts of the kidney, respond to a conventional treatment for canine leishmanosis. In addition, it could serve a basis to identify new potential markers that could be used for treatment monitoring of this disease.

## Methods

### Animals and sampling

This was a prospective multicenter study in which three clinics of southern Spain were involved lasting from November 2014 to May 2015.

The inclusion criteria for the dogs were: (1) diagnosis of canine leishmaniosis based on compatible clinical and/or laboratory changes [5] and the presence of high antibody titers (sample-to-positive ratio >1,1 calculated by OD sample/OD Low Positive Control) against *Leishmania* (Leiscan® Leishmania ELISA Test, Laboratorio Dr. Esteve S.A., Spain) and also positive on either cytology or real-time PCR from bone marrow or lymph node to detect the presence of the parasite; (2) absence of canine heartworm, *Anaplasma phagocytophylum*, *Borrelia burgdorferi*, and *Ehrlichia canis* antibodies using a fast test (Canine SNAP 4Dx, IDEXX laboratories); (3) inactive urine sediment; (4) not presenting with diseases other than leishmaniosis that could cause glomerulopathy; (5) being at stage I of the IRIS staging of CKD, with serum creatinine <1.4 mg/dl and having proteinuria (UPC > 0.5). Animals were excluded if they had been treated with angiotensin-converting enzyme inhibitors or any other drug in the 6 months prior to entering the study.

The dogs were evaluated at initial diagnosis and after 4 weeks of treatment with N-methylglucamine antimoniate (MGA) (50 mg/kg SC, two times daily) and allopurinol (10 mg/kg PO BID). A clinical score based on the severity of various clinical signs following a previous reported criteria [12] was obtained at diagnosis and after 4 weeks of treatment.

Blood samples were collected from the cephalic vein, placed in tubes containing a clotting activator, allowed to clot at room temperature, and centrifuged (3000 g, 10 min) to obtain serum and were stored at –80 °C until analysis. Urine samples were obtained by cystocentesis (5 mL, 22-G needles) and centrifuged (300 g, 2 min). Sediment was analyzed to exclude urine samples with active sediment and the supernatant were stored at −80 °C until analysis.

In addition, in order to obtain values of the different urinary biomarkers in healthy dogs, blood and urine was obtained from 10 healthy entire adult dogs. This group of dogs was integrated by 7 males and 3 females of different breeds with ages between 2 and 14 years, that come to the clinics for routine health controls or vaccination. All dogs show values of the hemogram and biochemical profile inside the reference range of the laboratory and were negative when serum was analyzed with the leishmania ELISA test.

The experimental procedure was approved by the Animal Experimentation Committee of the University of Murcia.

### Group allocation

Based on the evolution of the UPC after treatment, dogs were allocated into two groups: dogs that showed proteinuria (UPC > 0.5) before treatment and whose proteinuria increased or did not decrease after treatment (Group 1) and dogs that showed proteinuria (UPC > 0.5) before treatment and whose proteinuria decreased with treatment (Group 2).

### Urine biomarkers analysis

Protein, creatinine, ferritin and CRP in urine were measured by previously described methods [9, 13].

Automated assays using commercial kits were used for IgG and GGT (Beckman Coulter, Inc., Brea, CA, USA) and RBP and NAG (Dyazime, Poway, CA, USA) measurements. Analyses were performed in an automatic analyzer (Olympus AU400, Hamburg, Germany). All four assays when validated in urine showed inter and intra-assay imprecision lower than 15% and linearity under dilution resulted in linear regression equations with correlation coefficients (R^2^) higher than 0.98 in all cases.

SDMA was measured by liquid chromatography-mass spectrometry (LC-MS) following a previously described method [14]. The LC-MS system consisted of an Agilent 1100 Series HPLC (Agilent Technologies, Santa Clara, CA, USA) equipped connected to an Agilent Ion Trap XCT Plus Mass Spectrometer (Agilent Technologies, Santa Clara, CA, USA) using an electrospray interface (ESI). This assay showed an inter and intra-assay imprecision lower than 4%, high linearity with serial dilutions (R^2^ > 0.98) and recoveries after spiking different concentrations of SDMA to a canine serum were 96–103%.

### Statistical analysis

Kolmogorov-Smirnov test was performed to assess the normality of data, giving a nonparametric distribution. Data were then log-transformed and paired *t* test was used to compare values before and after treatment using a statistical program (Graph Pad Prism v.6 for Windows). Correlations between all parameters studied were determined using the Spearman correlation analysis. A *P* value of <0.05 was considered statistically significant.

## Results

Eighteen dogs naturally infected with *Leishmania infantum* of different breeds, ages (range, 2 to 13 years) and sex (13 males and 5 females all entire) were included in this study. Results of clinical scores are presented in Table 1. Initially all dogs included in the study had clinical and laboratory signs compatible with leishmaniosis, which were significantly reduced in all cases in response to treatment with the exception of 2 animals in the group 1: one dog initially had skin lesions and uveitis and maintained these clinical signs and one dog initially had skin lesions that worsened after treatment.

**Table 1:** Median, (25th and 75th percentiles) and percentage of median deviation between initial and follow-up samples of clinical scoring and the biomarkers recommended by IRIS. Group 1: dogs that showed proteinuria (UPC > 0.5) before treatment which did not decrease or even increased after treatment; Group 2: dogs that showed proteinuria (UPC > 0.5) before treatment which decreased after treatment

Parameter	Group 1 (*n* = 6)			Group 2 (*n* = 12)			Reference values at authors laboratory
	Inital	Follow up	% median deviation	Inital	Follow up	% median deviation	
Clinical Scoring	7 (6–8.2)	4 (2.5–4.2)	−42	9 (3–15)	2 (0.5–7)	−77	
UPC	1.492 (0.669–5.401)	2.270 (0.817–6.815)	52.14	1.318 (0.830–3.087)	0.432 (0.215–1.038)***	−67.22	<0.2
sCr (mg/dL)	0.88 (0.55–1.04)	0.92 (0.55–1.06)	1.04	0.69 (0.51–0.92)	0.94 (0.66–1.09)	13.62	<1.4
sSDMA (μg/dL)	10.40 (6.67–20.51)	9.91 (7.47–12.33)	−9.52	10.95 (7.85–16.35)	11.15 (8.57–12.18)	1.01	<14

***P < 0.001 compared to baseline

Results for the biomarkers recommended by the IRIS group, glomerular biomarkers and tubular biomarkers are presented in Tables 1, 2 and 3 respectively. No significant differences were found in any of the analytes when mean values of the two groups before the treatment were compared.

**Table 2:** Median, (25th and 75th percentiles) and percentage of median deviation between initial and follow-up samples of biomarkers of glomerular damage. Group 1: dogs that showed proteinuria (UPC > 0.5) before treatment which did not decrease or even increased after treatment; Group 2: dogs that showed proteinuria (UPC > 0.5) before treatment which decreased after treatment

Parameter	Group 1 (*n* = 6)			Group 2 (*n* = 12)			Control dogs (*n* = 10)
	Inital	Follow up	% median deviation	Inital	Follow up	% median deviation	
uIgG/Cr (mg/g)	202.7 (31.1–4986.0)	81.5 (3.5–3603.0)	-24.8	176.1 (36.6–2996.0)	40.4 (12.9–408.4)**	−77.02	0.01 (0.008–0.018)
uCRP/Cr (μg/g)	6.06 (1.87–29.1)	8.38 (1.78–16.1)	38.2	15.40 (4.624–74.67)	3.21 (2.48–5.09)**	−79.16	1.03 (0.74–1.89)
uFerr/Cr (μg/g)	27.1 (5.6–45.9)	31.5 (5.2–60.5)	16.2	23.48 (8.95–60.92)	13.71 (5.35–30.57)*	−41.61	2.00 (0.58–3.44)

**P<0.01 compared to baseline, *P<0.05 compared to baseline

**Table 3:** Median, (25th and 75th percentiles) and percentage of median deviation between initial and follow-up samples of biomarkers of tubular damage. In Group 1: dogs that showed proteinuria (UPC > 0.5) before treatment which did not decrease or even increased after treatment; Group 2: dogs that showed proteinuria (UPC > 0.5) before treatment which decreased after treatment

Parameter	Group 1 (*n* = 6)			Group 2 (*n* = 12)			Control dogs (*n* = 10)
	Inital	Follow up	% median deviation	Inital	Follow up	% median deviation	
uRBP/Cr (mg/g)	0.880 (0.049–7.453)	0.506 (0.173–8.608)	−42.5	0.484 (0.227–2.650)	0.169 (0.037–0.502)*	−65.08	0.21 (0–0.99)
uNAG/Cr (UI/g)	18.49 (4.35–98.82)	18.30 (5.64–28.57)	−1.03	14.77 (5.31–28.32)	6.20 (3.84–14.56)*	−58.02	7.97 (5.27–11.19)
uGGT/Cr (UI/g)	31.3 (20.0–101.8)	28.4 (9.9–95.2)	−9.26	15.38 (11.05–90.36)	10.78 (7.44–19.63)	−29.91	10.99 (8.37–18.87)

*P<0.05 compared to baseline

In group 1, biomarkers recommended by IRIS group (UPC, sCr and sSDMA) did not show statistically significant changes after treatment. In addition, no significant changes were observed in biomarkers of glomerular and tubular damage.

In group 2, of the biomarkers recommended by IRIS group, only UPC showed a significant decrease (67.22%) after treatment. All biomarkers of glomerular damage showed a significant decrease after treatment that was more significant and of higher magnitude in the case of urinary immunoglobulin G to creatinine ratio (uIgG/Cr) (77.02%) and urinary C-reactive protein to creatinine ratio (uCRP/Cr) (79.16%). Regarding the biomarkers of tubular damage, urinary retinol-binding protein to creatinine ratio (uRBP/Cr) and urinary N-aceytyl-β-D-glucosaminidase to creatinine ratio (uNAG/Cr) showed a significant decrease after treatment (65.08 and 58.02% respectively) and urinary gamma glutamyl-transpeptidase (uGGT/Cr) did not show any significant change. Correlations between all analytes are depicted in Table 4. A positive significant association was found between UPC and uIgG/Cr, uCRP/Cr and uRBP/Cr.

**Table 4:** Correlations between all analytes of our study

	UPC	sCr	sSDMA	uIgG/Cr	uCRP/Cr	uFerr/Cr	uRBP/Cr	uNAG/Cr	uGGT/Cr
UPC		0.39	−0.09	0.75***	0.50*	0.42	0.59**	0.43	0.35
sCr	0.39		0.12	0.39	−0.11	0.11	0.42	0.23	−0.22
sSDMA	−0.09	0.12		0.15	−0.13	−0.29	−0.38	−0.04	−0.38
uIgG/Cr	0.75***	0.39	0.15		0.08	0.31	0.47*	0.12	0.08
uCRP/Cr	0.50*	−0.11	−0.13	0.08		0.69***	0.54*	0.48*	0.31
uFerr/Cr	0.42	0.11	−0.29	0.31	0.69***		0.63**	0,53*	0.53*
uRBP/Cr	0.59**	0.42	−0.38	0.47*	0.54*	0.63**		0.14	0.27
uNAG/Cr	0.43	0.23	−0.04	0.12	0.48*	0,53*	0.14		0.33
uGGT/Cr	0.35	−0.22	−0.38	0.08	0.31	0.53*	0.27	0.33	

* *P* <0.05

** *P* <0.01

*** *P* <0.001

## Discussion

This study reports the changes in a panel of various kidney biomarkers in dogs with leishmaniosis after treatment with MGA and allopurinol. Biomarkers included those recommended by the IRIS, and additional urinary glomerular and tubular biomarkers previously used for kidney evaluation in the dog. Two of the biomarkers recommended by the IRIS, namely sCr and UPC, were used as inclusion criteria for the dogs, since only dogs with sCr lower than 1.4 mg/dL and UPC higher than 0.5 were included in the study. Therefore we selected dogs that had proteinuria but no azotemia, being in stage I according with the IRIS staging of CKD. This allowed us to eliminate azotemia as a confounding factor and focus our study to those biomarkers that could better reflect and correlate with the evolvement of the proteinuria.

The evolution of UPC after treatment was used to divide the dogs in two groups: dogs that showed a decrease in UPC and dogs that did not show a decrease in UPC after treatment. Therefore the dynamics of the analytes in the different possible unfolding of UPC in dogs with leishmaniosis after treatment were compared. The existence of dogs that do not show an improvement in UPC after a conventional treatment for canine leishmaniosis has been previously described [15]. In our study we found four of the six dogs in group 1 that showed an improvement in clinical signs despite experiencing an increase in UPC. This is in agreement with previous studies that did not found a relationship between clinical signs and UPC in dogs with leishmaniosis, with non-proteinuric dogs showing clinical signs [16] and also with the personal observation of the authors of the presence of dogs with evident proteinuria but without clinical signs.

sSDMA, which is a biomarker recently recommended by the IRIS group, did not show significant changes after treatment in dogs that showed a decrease in UPC. Therefore, although SDMA is considered as one of the most sensitive serum biomarker to evaluate the glomerular filtration rate [14] and it was increased in some dogs in this study (considering 14 μg/dL as a cut-off point), its use for monitoring the evolution of proteinuria after treatment in dogs with leishmaniosis in IRIS stage I would be questionable. This would be in line with a previous report in dogs in which changes in proteinuria appeared before changes in SDMA [17]. Probably the SDMA would be a better predictor of changes in creatinine values, as has been previously described [14], than variations in proteinuria. However, our study used a relatively low number of dogs and further studies with a larger number of dogs should be made to corroborate our findings.

When other glomerular biomarkers in addition to UPC were studied (uIgG/Cr, uCRP/Cr and urinary ferritin to creatinine ratio (uFerr/Cr)), all of them had a significant decrease in the dogs that reduced the UPC after treatment. uCRP/Cr and uIgG/Cr showed a higher percentage of decrease after treatment than UPC, and uIgG/Cr showed the highest correlation with UPC of all the biomarkers studied. In a recent study, uIgG was found to be highly positively correlated with CKD caused by immune complex-mediated glomerulonephritis (ICGN) [18], which is the most common kidney lesion produced in leishmaniosis. These authors found that although its sensitivity was lower, uIgG showed higher specificity than UPC for detecting ICGN. In addition uIgG was highly associated, as UPC did, with ultrastructural glomerular damage evaluated by transmission electron microscopy. In our study, the glomerular markers did not show significant changes in the dogs with no decrease in UPC after treatment. Although in general the data of group 1 due to the low number of dogs should be taken with caution, we observed in 3 cases that uIgG/Cr and uCRP/Cr decreased after treatment although UPC did not decrease. Further studies should be undertaken the elucidate the possible explanation of this finding.

Two tubular markers (uRBP/Cr and uNAG/Cr) showed a significant decrease in dogs with reduced UPC, and in the case of uRBP/Cr the magnitude of this decrease was similar to UPC, being uRBP/Cr and UPC highly associated. uRBP is described as a particularly promising marker of CKD progression since it strongly correlates with histologic lesions at glomerular and tubular level [19]. None of the tubular markers showed any significant changes in the dogs with no reduction in UPC after treatment. Although traditionally considered as tubular markers, uRBP/Cr and uNAG/Cr are also moderately to strongly correlated with glomerular lesions [18, 19] and even uNAG/Cr is strongly correlated with glomerular damage but not with tubular damage in chronic proteinuric nephropathy [18]. On the other hand, the lack of a significant decrease in uGGT/Cr found in the dogs that reduced UPC, could be explained by the fact that this analyte is more related with tubular dysfunction [11].

We did not characterize if the proteinuria of the dogs was glomerular, tubular or mixed –which should be considered as a limitation of our study-. However it could be hypothesized that uRBP/Cr and uNAG/Cr are increased in our study because they are correlated with glomerular damage. Additionally a tubular dysfunction could exist in the dogs of our study. This would be in line with the findings that tubular damage commonly occurs concurrently with glomerular damage in cases of chronic kidney disease, and that in these cases damage to one compartment of the kidney affect the other [18]. In cases of glomerulonephritis, proteinuria develops which can result in tubular damage. In fact, it has been reported that most dogs affected with leishmaniosis have mixed (glomerular and tubular) proteinuria [11]. Another cause for the tubular dysfunction could be related with the possibility of MGA to produce tubular damage [20]. Further studies should be undertaken to elucidate the mechanisms leading to tubular disfunction in canine leishmaniosis and its evolution during treatment.

It is important to point out that this is a pilot study and it has major limitations, such as the low number of animals studied and the lack of histological analysis to characterize the type of lesion of the dogs and to allow studying the sensitivity and specificity of the different biomarkers to detect and monitor kidney damage.

## Conclusions

In dogs with leishmaniosis at IRIS stage I that reduced UPC after treatment, there were no significant changes in serum creatinine and sSDMA. However all the urine biomarkers evaluated with the exception of GGT/Cr showed a significant decrease. These decreases were more evident in those markers related with glomerular function, being uIgG/Cr the biomarker more associated with UPC. Further studies involving a larger number of animals and histological analysis of the kidney would be recommended to corroborate these findings and evaluate the routine practical use of these urine biomarkers for the early recognition of kidney damage in dogs with canine leishmaniosis and for monitoring kidney status during therapy.

## Abbreviations

CKD: Chronic kidney disease
CRP: C-reactive protein
GGT: Gamma glutamyl-transpeptidase
ICGN: Immune complex-mediated glomerulonephritis
IgA: Immunoglobulin A
IgG: Immunoglobulin G
IRIS: International Renal Interest Society
LC-MS: Liquid chromatography-mass spectrometry
MGA: N-methylglucamine antimoniate
NAG: N-aceytyl-β-D-glucosaminidase
RBP: Retinol-binding protein
sCr: Serum creatinine
SDMA: Symmetric dimethylarginine
sSDMA: Serum symmetric dimethylarginine
uCRP: Urinary C-reactive protein
uCRP/Cr: Urinary C-reactive protein to creatinine ratio
uferritin: Urinary ferritin
uGGT: Urinary gamma glutamyl-transpeptidase
uIgG: Urinary immunoglobulin G
uIgG/Cr: Urinary immunoglobulin G to creatinine ratio
uNAG: Urinary N-aceytyl-β-D-glucosaminidase
uNAG/Cr: Urinary N-aceytyl-β-D-glucosaminidase to creatinine ratio
UPC: Urinary protein to creatinine ratio
uRBP: Urinary retinol-binding protein
uRBP/Cr: Urinary retinol-binding protein to creatinine ratio
